# Effectiveness of Community-Delivered Functional Power Training Program for Frail and Pre-frail Community-Dwelling Older Adults: a Randomized Controlled Study

**DOI:** 10.1007/s11121-021-01221-y

**Published:** 2021-03-19

**Authors:** Nien Xiang Tou, Shiou-Liang Wee, Wei Ting Seah, Daniella Hui Min Ng, Benedict Wei Jun Pang, Lay Khoon Lau, Tze Pin Ng

**Affiliations:** 1 Geriatric Education and Research Institute (GERI), 2 Yishun Central 2, Tower E Level 4 GERI Admin, Singapore, 768024 Singapore; 2 Health and Social Sciences Cluster, Singapore Institute of Technology, Singapore, Singapore; 3 Graduate Medical School, Program of Health Services and System Research, Duke-National University of Singapore, Singapore, Singapore; 4 Department of Psychological Medicine, National University of Singapore, Singapore, Singapore

**Keywords:** Exercise, Frailty, Physical function, Senior activity centers, Successful aging

## Abstract

Translation of community-based functional training for older adults to reduce frailty is still lacking. We evaluated the effectiveness and implementation of a community-delivered group-based functional power training (FPT) program for frail older adults within their neighborhoods. A two-arm, multicenter assessor-blind stratified randomized controlled trial was conducted at four local senior activity centers in Singapore. Sixty-one community-dwelling older adults with low handgrip strength were randomized to intervention (IG) or control (CG) group. The IG underwent the FPT program (power and balance exercises using simple equipment) delivered by a community service provider. The 12-week program comprised 2 × 60 min sessions/week. CG continued usual activities at the centers. Functional performance, muscle strength, and frailty status were assessed at baseline and 3 months. Program implementation was evaluated using RE-AIM framework. The program was halted due to Coronavirus Disease 2019-related suspension of senior center activities. Results are reported from four centers, which completed the program. IG showed significantly greater improvement in the Short Physical Performance Battery test as compared to CG (*p* = 0.047). No effects were found for timed up and go test performance, muscle strength, and frailty status. The community program exhibited good reach, effectiveness, adoption, and implementation. Our study demonstrated that FPT was associated with greater improvement in physical function in pre-frail/frail participants as compared to exercise activities offered at local senior activity centers. It is a feasible intervention that can be successfully implemented for frail older adults in their neighborhoods.

*Trial registration* ClinicalTrials.gov, NCT04438876. Registered 19 June 2020–retrospectively registered.

**Supplementary Information:**

The online version contains supplementary material available at 10.1007/s11121-021-01221-y.

## Background

Community-dwelling older adults are prone to developing frailty (Ofori-Asenso et al., 2019). Frailty has been defined as “a clinical state in which there is an increase in an individual’s vulnerability for developing an increased dependency and/or mortality when exposed to a stressor” (Morley et al., 2013). It can occur before 60 years of age and the onset escalates in those aged 70 years and older (Hoogendijk et al., 2018). Frailty is more common in persons with lower education and/or socio-economic status (Wei et al., 2017). Furthermore, physically frail individuals with low socio-economic status and social support are more prone to suffer functional disability (Teo et al., 2017).

Frailty is not a contraindication to exercise but a reason to prescribe it. Frailty can be delayed or reversed through interventions where physical training is a key component, especially during the pre-frail stage (Apóstolo et al., 2018; Liu et al., 2019; Travers et al., 2019). There is wide consensus for pre-frail or frail older adults to be offered a physical activity program with a progressive, resistance training component (Dent et al., 2019; Fragala et al., 2019).

Resistance training typically engages low-velocity contractions at 50–80% of maximal strength. In contrast, power training is characterized by performing the concentric phase (force production by shortening of muscles) at high velocity (as far as possible) with a slow eccentric phase (force production by lengthening of muscles) to achieve the greatest benefit of muscular power and strength. Such training is effective and has emerged as an alternative modality of resistance training to preserve function of older persons to perform activities of daily living that often require quick, forceful motions (Ramírez-Campillo et al., 2014). Randomized controlled trials have demonstrated that high velocity resistance training could improve physical function among pre-frail (Zech et al., 2012) and frail individuals (Cadore et al., 2014). Given the high local prevalence of pre-frailty (45%) and frailty (5%) in community-dwelling older adults (Wei et al., 2017), it is imperative for research efforts to move beyond laboratory-based efficacy trials to evaluate effectiveness in real-world settings, with a focus on implementation and real-world partnerships.

Effective interventions in research settings are often not successfully translated to practice due to poor external validity (Glasgow et al., 2003). For example, the requirement of specialized equipment and facility in the aforementioned power training exercise interventions (Cadore et al., 2014; Ramírez-Campillo et al., 2014; Zech et al., 2012) can limit the translation of these programs in community settings that often do not have access to such facilities and equipment. To bridge such a research-practice gap, efficacy of interventions needs to be examined in targeted population settings to better understand the enablers and challenges of the intervention in actual practice (Peters et al., 2013).

We had previously shown the feasibility and potential of a small group functional power training (FPT) program using bodyweight, resistance bands, and chairs for frail older adults at a local senior activity center (Tan et al., 2018). However, this finding needs to be replicated in a larger study at multiple sites to ascertain the effectiveness of the intervention for wider translation. Therefore, the purposes of this study were to examine the effectiveness and evaluate the implementation of an FPT exercise program for pre-frail and frail community-dwelling older adults through local senior activity centers. These centers are located at ground level of residential apartments of public housing and are accessible to older adults with poorer socio-economic status and low social support. Fried et al. (2001) frailty criteria were adopted, with muscle weakness as an inclusion criterion. It was hypothesized that the program implemented in the local residential community would be effective to improve functional performance and reduce frailty for individuals with low muscle strength.

## Methods

### Study Design and Participants

A two-arm, multicenter assessor-blind stratified randomized controlled trial among pre-frail and frail community-dwelling older adults was conducted between March 2019 and February 2020 (ClincalTrials.gov Identifier: NCT04438876). Seven senior activity centers located within the residential estates in Singapore were approached, and the study was conducted at four centers. To be eligible for the study, participants had to (a) be aged 55 years and older, (b) have low muscle strength, (c) ambulate without human assistance and have no other physical limitations to participation and adherence to exercise, and (d) be able to understand basic instructions. Low muscle strength was defined as handgrip strength (HGS) less than 26 kg and 18 kg in men and women, respectively, according to the Asian Working Group for Sarcopenia (AWGS) 2014 consensus (Chen et al., 2014). Participants were excluded if they (a) were currently enrolled in another study, (b) had any acute musculoskeletal injury or other contraindication to exercise, (c) were unable to participate in the full duration of the study, (d) were unwilling to participate if not assigned to the intervention group, and (e) were deemed not suitable to participate in exercise by a medical doctor. After initial eligibility screening by senior activity center staff or research coordinator, a doctor examined each subject for medical clearance prior to exercise participation and excluded subjects who did not meet the medical criteria. Participants enrolled at each site were then randomly allocated to either the control group (CG) or the intervention group (IG) with a 1:1 allocation ratio based on computerized block randomization with randomly selected block sizes of 4 or 6. Participants were stratified according to their gender group and HGS (< 18 kg for males, < 10 kg for females). Research staff ensured that there was no treatment contamination between intervention and control participants. Ethical approval was obtained from the National Healthcare Group DSRB (2018/00593). All participants provided written informed consent prior to participation in the study. Reporting of this study was in accordance with the “CONSORT” statement (see supplementary material).

### Intervention Group

The IG underwent a 12-week structured FPT program, conducted by an exercise-science qualified trainer from a community service provider "(Empower Ageing Limited, https://empower.org.sg/frailty-solutions/). Hourly sessions were held twice weekly at local senior activity centers. The exercise intervention comprised progressive power and balance exercises that targeted both upper and lower body muscles. The list of specific exercises for each respective session is presented in Table 1 and detailed elsewhere (Empower Ageing Limited, https://empower.org.sg/frailty-solutions/). Participants performed three sets of each power exercise, with 10–20 repetitions per set and 12–60 repetitions per set for balance exercises. For the power training, body weight and/or resistance bands were used as resistance and participants were instructed to move as fast as they can during the concentric phase and slowly during the eccentric phase (approximately 3 s) of the exercise movements. To ensure safety, blood pressure of participants was measured before and after each session with an automated sphygmomanometer (Omron HEM-7121, Omron, Kyoto, Japan). Arterial blood oxygen saturation and heart rate were measured at start, mid-point, and end of each exercise session with pulse oximetry (MD300C63, ChoiceMMed, Bristol, Pennsylvania, USA) to monitor exercise intensity levels. Participants with either (a) blood oxygen levels below 95% saturation; (b) high resting heart rate (≥ 90 beats per minute); (c) abnormal resting blood pressure (systolic blood pressure ≥ 130 mmHg or diastolic blood pressure ≥ 80 mmHg); (d) giddiness or (e) any form of discomfort did not initiate or continue with training session. Participants also rated their perceived exertion after each exercise set and were encouraged to alert the trainer if they felt any discomfort during the training. We monitored and recorded adverse events throughout the intervention program. In addition, participants also received monthly health education talks on nutrition and cognition, conducted by staff from Geriatric Education and Research Institute.

**Table 1:** 12-Week functional power training intervention program

Components	Wk 1–Wk 2	Wk 3–Wk 4	Wk 5–Wk 6	Wk 7–Wk 8	Wk 9–Wk 10	Wk 11–Wk 12
Lower-body power	• Sit to stand	• Sit to stand^a^	• Squats	• Sit to stand^a^	• Sit to stand plus swap seats^a^	• Squats plus press
	• Standing knee ups	• Hip extension	• Hip extension^a^	• Standing knee ups^a^	• Seated knee extension^a^	• Hip extension^a^
	• Standing calf raises	• Standing toe up	• Calf raises with toe ups	• Hip abduction	• Calf raises with toe ups	• Standing knee ups^a^
		• Seated heel drag^a^		• Seated heel drag^a^		
				• Knee flexion and hip extension^a^		
Upper-body power	• Bicep curl^a^	• Chest press^a^	• Seated low row^a^	• Chest press^a^	• Bicep curl^a^	• Standing row^a^
				• Shoulder press^a^		• Shoulder press^a^
Balance and mobility	• Tandem balance	• Side-to-side plus reach	• Clock tapping	• Speed plus zigzag walk relay		• Quick feet
	• Clock tapping	• Marching with side step	• Mini lunges		• Quick feet	• Speed walk
	• Side reach	• Tandem walk	• Speed walk		• Mini lunges	• Crossing creek
	• Zigzag walk		• Side step			• Farmer's walk
			• Tandem walk			

^a^Exercise performed with resistance bands

### Control Group

Participants in the CG could continue with the available exercise program at the respective senior activity centers. Senior activity centers typically had chairs arranged for participants who followed daily video of stretching, aerobic, and balance exercises produced by the Health Promotion Board (Health Promotion Board, 2017). The CG was given an exercise manual with the list of exercises in the intervention program. CG participants were also encouraged to attend the health education talks. Adherence of CG participants to center activity was not monitored.

### Measurements

Objective physical outcome assessments were conducted at baseline and 3-month follow-up by assessors who were blinded to participants’ group allocation.

#### Physical Function Assessment

Physical function assessments included HGS (Rantanen et al., 1999), knee extensor strength (KES) (Guralnik et al., 1995), timed up and go (TUG) (Shumway-Cook et al., 2000), and the Short Physical Performance Battery (SPPB) (Mijnarends et al., 2013). As a standardized measurement of muscular strength among older adults (Roberts et al., 2011), HGS was assessed using the Jamar Plus + Digital Hand Dynamometer (Patterson Medical, Evergreen Boulevard, Cedarburg, USA). KES was measured using a spring gauge strapped 10 cm above the ankle joint, and the highest of four readings (two trials per leg) recorded. For TUG, participants stood up from a chair, walked 3 m and back, then sat back down. The test was performed twice, and the average time recorded. SPPB comprised three components: balance, gait speed, and repeated chair stands. A composite score of 0–12 points was calculated (Guralnik et al., 1994), whereby higher scores indicate better functional performance.

#### Frailty Status Assessment

Frailty status of participants was assessed using Fried’s Frailty Criteria, which characterizes frailty based on five components: weakness, unintentional weight loss, slowness, exhaustion, and low physical activity (Fried et al., 2001). Weakness was identified using the AWGS 2014 criteria as HGS less than 26 kg for men and 18 kg for women (Chen et al., 2014). Unintentional weight loss was defined by either BMI less than 18.5 kg/m^2^ or self-reported unintentional weight loss of at least 4.5 kg (10 lb) in the last 6 months. Slowness was determined by 6 m walking speed with specified cut-offs based on gender and height. Exhaustion was self-reported through a 3-item questionnaire adapted from the SF-12 survey (Ware Jr et al., 1996). The Longitudinal Aging Study of Amsterdam Physical Activity Questionnaire was administered to assess participants’ physical activity levels (Stel et al., 2004). Low physical activity was defined as energy expenditure less than 383 kcal/week and 270 kcal/week for men and women, respectively. One point was given for presence of each component, and frailty status classification was defined as robust (0), pre-frail (1–2), and frail (3–5) (Fried et al., 2001).

#### Other Measures

All participants answered a questionnaire on their baseline demographic information such as age, gender, housing type, and smoking history. Participants also self-reported whether they were diagnosed by a doctor for a list of specific medical conditions. Anthropometric measurements such as height, weight, body mass index (BMI), and waist circumference were taken during the baseline assessment session. The Mini-Mental State Examination was administered to assess cognitive function in participants. It was scored out of 30, with higher scores indicating better cognitive function (Folstein et al., 1975).

### Evaluation of Program Implementation

The implementation of the FPT intervention was evaluated using the RE-AIM framework (Glasgow et al., 1999), a model designed to appraise public health interventions. Present study employed four of the five dimensions specified in the framework: reach, effectiveness, adoption, and implementation. First, “reach” was calculated as the percentage of eligible participants who enrolled in the study. Second, “effectiveness” was assessed based on objective outcome assessments stated above, program attrition rate, and participant experience. Participant experience was reported post-intervention in the IG only using a 9-item questionnaire administered by the research team. Participants responded by indicating the extent of agreement with the questionnaire items on a 5-point Likert scale ranging from 1 (strongly disagree) to 5 (strongly agree). Third, “adoption” was determined as proportion of senior activity centers approached that agreed to implement the intervention. Fourth, “implementation” was assessed based on fidelity of intervention delivery at both provider and participant levels. At the provider-level, the extent of deviation between the trainer’s delivery and the intended exercise program was recorded by a research coordinator who observed every training session and recorded attendance. At the participant-level, implementation was evaluated by program attendance rate and proportion of the exercise program completed by participants. The intervention was deemed to have good adherence if a mean attendance rate of at least 80% was achieved at the participant level. The dimension of “maintenance” was excluded since the present study was not designed to determine the longer-term effects of the intervention. We monitored and recorded adverse events throughout the course of the program.

### Sample Size Calculation

Based on a priori power analysis (G*Power 3.1.9.3) using a power of 0.90 and error probability of 0.05, a sample size of 50 participants is required for each group to detect an assumed 20% difference in SPPB between IG and CG. In addition, with an assumption of 15% dropout rate, a sample size of 120 participants was initially targeted.

### Statistical Analysis

Differences in baseline measures between CG and IG were compared using independent sample *t* tests for continuous variables and chi-squared tests for categorical variables.

Levene’s test was conducted to ensure no violation of equal variance assumption. Linear mixed-effect modeling was performed to examine changes in physical function and frailty status between baseline and 3-month follow-up across both groups. Primary independent variables in each model included treatment groups, time, as well as group × time interaction. The models included random intercepts to account for correlations between repeated measures for each participant and were adjusted for age, gender, and physical activity levels. Clustering effect at the center level was omitted as intercepts did not vary significantly across centers. Data analyses were performed based on the intention-to-treat principle (Moher et al., 2001), and the maximum likelihood method was employed to impute missing values. Post hoc pairwise comparisons were conducted to examine the main effect of time in respective groups. Statistical significance level was set at 0.05, and all analyses were performed using Statistical Package for the Social Science (SPSS Version 20.0, Chicago, IL). Threshold values were selected as standardized effect size (ES) of mean differences and deemed as 0.2, 0.6, 1.2, and 2.0 for small, moderate, large, and very large, respectively (Hopkins et al., 2009).

## Results

**Fig. 1:**
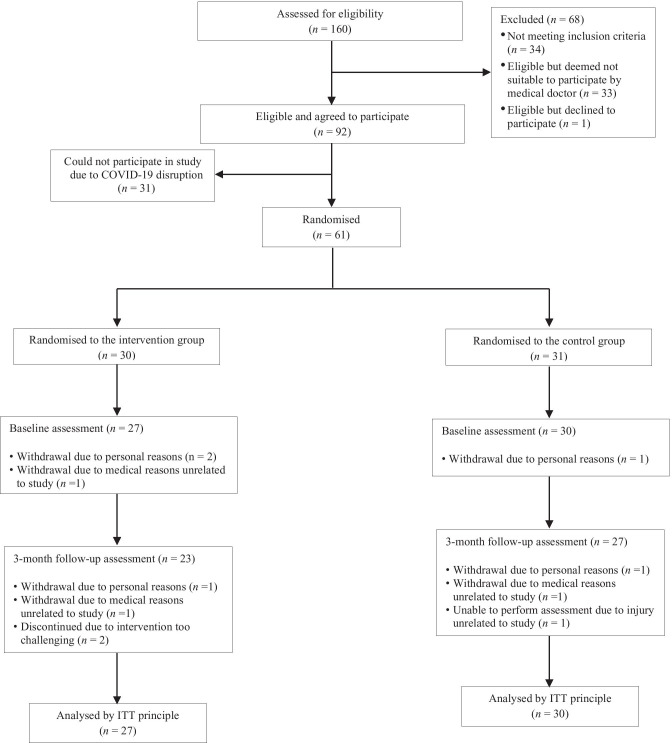
Study flow diagram. ITT, intention-to-treat

Figure 1 presents the study flow from screening to analyses. A total of 110 participants were screened for eligibility through partnership with four senior activity centers in this study, of which 61 were randomized into either the CG (*n* = 31) or IG (*n* = 30). In partnership with a fifth senior activity center, another 50 individuals were screened, 33 completed pre-participation medical examination, and 31 eligible signed informed consent on 4 Feb 2020. The first case of Coronavirus Disease 2019 (COVID-19) in Singapore was confirmed on 23 January 2020. We could not proceed with the study after health authority mandated suspension of all group senior activities with effect from 8 Feb 2020 and the decision was made in May 2020 (with suspension still in force) to close the study and perform analyses on the 61 participants from the four centers. Post hoc power analysis revealed that this sample size was adequate to detect effects of moderate size. Four participants in the CG and seven participants in the IG did not complete the study. Table 2 presents the descriptive characteristics of both CG and IG. No significant differences were found in baseline measures between the two groups.

**Table 2:** Baseline characteristics of participants in both CG and IG

	Control group (*n* = 30)	Intervention group (*n* = 27)	*p*
Age (years)	71.53 ± 7.99	72.07 ± 8.14	0.802
Gender, female	28 (93.3%)	25 (92.6%)	1.000
Housing, < 3 rooms apartment	17 (56.7%)	12 (44.4%)	0.448
BMI (kg/m^2^)	23.45 ± 3.59	25.30 ± 5.44	0.141
WC (cm)	83.68 ± 11.00	86.41 ± 11.62	0.369
MMSE	26.30 ± 3.22	25.48 ± 2.87	0.315
PA (kcal/week)	3448.80 ± 2613.28	3835.37 ± 2410.26	0.564
Smoking history^a^			0.587
Current smoker	1 (3.3%)	0 (0.0%)	
Ex-smoker	3 (10.0%)	2 (7.4%)	
Non-smoker	26 (86.7%)	25 (92.6%)	
CVD	0 (0.0%)	2 (7.4%)	0.426
Hypertension	21 (70.0%)	17 (63.0%)	0.778
High cholesterol	18 (60.0%)	15 (55.6%)	0.944
Osteoporosis	7 (23.3%)	4 (14.8%)	0.633
Osteopenia	4 (13.3%)	2 (7.4%)	0.768
Stroke	0 (0.0%)	2 (7.4%)	0.426
Diabetes	8 (26.7%)	7 (25.9%)	1.000
Arthritis	15 (50.0%)	10 (37.0%)	0.473
Frailty status			0.958
Pre-frail	30 (100.0%)	26 (96.3%)	
Frail	0 (0.0%)	1 (3.7%)	

Data presented in *n* (%) or mean ± SD

*BMI* body mass index, *WC* waist circumference, *MMSE *mini-mental state examination, *PA* physical activity, *CVD* cardiovascular disease

^a^Smoking history was self-reported

### Effectiveness of Program Intervention

**Fig. 2:**
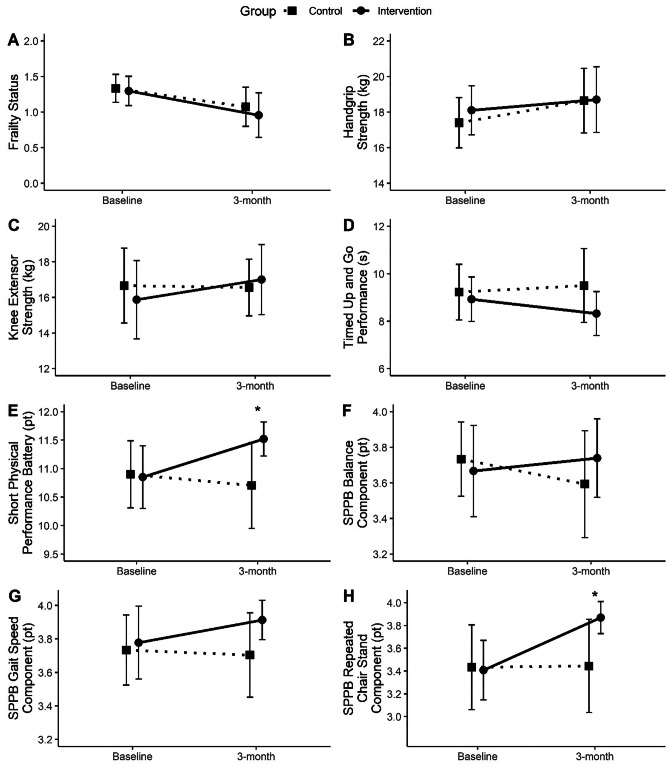
Outcome measures at baseline and 3-month follow-up across groups. **a** Frailty status; **b** handgrip strength; **c** knee extensor strength; **d** timed up and go performance; **e** short physical performance battery; **f** SPPB balance component; **g** SPPB gait speed component; **h** SPPB repeated chair stand component. *Significant group-time interaction (*p* < 0.05). Data presented in mean ± 95% CI

The outcome measures at baseline and 3-month follow-up are presented in Table 3 and Fig. 2. No significant differences were found in all outcome measures between CG and IG at baseline. Significant interaction between group and time was found for SPPB [*F*(1, 50.678) = 4.320, *p* = 0.043]. However, no significant interaction between group and time was found for frailty status *F*(1, 55.403) = 0.225, *p* = 0.637], HGS [*F*(1, 52.667) = 0.218, *p* = 0.642], KES [*F*(1, 52.846) = 1.038, *p* = 0.313], and TUG [*F*(1, 51.653) = 2.601, *p* = 0.113]. Among the components of SPPB, group × time interaction effect was found only for repeated chair stands [*F*(1, 52.471) = 4.204, *p* = 0.045].

**Table 3:** Outcome measures at baseline and 3-month follow-up across CG and IG

	Control group			Intervention group			
	Baseline (*n* = 30)	3 months (*n* = 27)	*p* value	Baseline (*n* = 27)	3 months (*n* = 23)	*p* value	Group-time interaction* p* value
Frailty status	1.33 ± 0.55	1.07 ± 0.73	0.034*	1.30 ± 0.54	0.96 ± 0.77	0.010*	0.637
HGS (kg)	17.40 ± 3.95	18.64 ± 4.81	0.020*	18.10 ± 3.65	18.70 ± 4.52	0.117	0.642
KES (kg)	16.66 ± 5.89	16.56 ± 4.22	0.928	15.87 ± 5.83	17.00 ± 4.82	0.197	0.313
TUG (s)	9.22 ± 3.27	9.50 ± 4.13	0.706	8.92 ± 2.49	8.32 ± 2.27	0.071	0.113
SPPB (pt)	10.90 ± 1.65	10.81 ± 2.00	0.937	10.85 ± 1.46	11.52 ± 0.73	0.008*	0.043*
Balance (pt)	3.73 ± 0.58	3.59 ± 0.80	0.169	3.67 ± 0.68	3.74 ± 0.54	0.622	0.195
Gait speed (pt)	3.73 ± 0.58	3.70 ± 0.67	0.938	3.78 ± 0.58	3.91 ± 0.29	0.327	0.439
Chair stand (pt)	3.43 ± 1.04	3.44 ± 1.09	0.628	3.41 ± 0.69	3.87 ± 0.34	0.002*	0.045*

Model adjusted for age, gender, and physical activity levels

Data presented in mean ± SD

*HGS* handgrip strength,* KES *knee extensor strength, *TUG *timed up and go, *SPPB* short physical performance battery

*p < 0.05

Post hoc pairwise comparisons found significant changes in outcome measures between baseline and 3-month follow-up in both groups. For the IG, post-program frailty status significantly improved by 0.36 points, 0.51 ES, 95% confidence interval (95CI) [0.09, 0.64], *p* = 0.010. Similarly, SPPB scores improved by 0.52 points (4.8%), 0.58 ES, 95CI [0.14, 0.90], *p* = 0.008. Among the SPPB components, only repeated chair stand component was found to significantly improve by 0.42 points, 0.85 ES, 95CI [0.158, 0.671], *p* = 0.002. Improvement of 0.58 s in TUG performance did not achieve statistical significance, 0.25 ES, 95CI [− 1.21, 0.05], *p* = 0.071. No significant differences were found for HGS and KES. For the CG, significant improvement between baseline and 3-month follow-up was found in frailty status by 0.28 points, 0.40 ES, 95CI [0.02, 0.53], *p* = 0.034; and HGS by 1.19 kg, 0.28 ES, 95CI [0.20, 2.17], *p* = 0.020. No significant changes were found in KES, TUG, and SPPB performance for the CG.

### Program Implementation Outcomes

#### Reach 

A total of 160 older adults were assessed for eligibility, of which 34 (21.3%) did not meet the study’s inclusion criteria. Out of the remaining 126 (78.7%), 33 (26.2%) were deemed not suitable to participate in the intervention by a medical doctor. Among the 93 eligible individuals, one (1.1%) declined to participate. Therefore, 98.9% of eligible individuals agreed to participate in the FPT training program. However, 31 participants could not start the intervention due to the study being disrupted by COVID-19.

#### Effectiveness 

The effectiveness of the intervention on objective physical outcome measures was reported above. Among the 27 participants who started the intervention, four (14.8%) did not complete the exercise program. One withdrew from the study due to personal reasons and one dropped out because of medical reasons unrelated to the study. There was no other adverse event during the program. Two participants reported that the exercise intervention was too challenging and dropped out on the fourth and fifteenth session, with attendance rate of 25% and 57.1%, respectively. The participant experience questionnaire was administered to 23 participants who completed the intervention. All participants agreed that the intervention was positive in terms of organization, engagement, and relevance to daily activities (Table 4). On average, participants also reported that they benefited from the intervention and felt happier. In addition, all participants indicated that they would participate in such an exercise program in the future. Only one participant disagreed that the intervention improved the social interaction among participants.

**Table 4:** Responses on intervention participant experience (*n* = 23)

Questionnaire items	Score
The exercise program was well structured, organized, and easy to follow	4.35 ± 0.49
The exercise program was fun, enjoyable and engaging	4.57 ± 0.59
The exercise program was relevant to my daily activities	4.13 ± 0.92
The exercise program helped to improve my social interaction with other participants	4.04 ± 0.82
After the exercise program, I feel more energetic and happy	4.35 ± 0.65
After the exercise program, I feel stronger and confident with daily activities	4.30 ± 0.56
I feel that I have benefited from the exercise program	4.52 ± 0.51
I will recommend this exercise program to others	4.48 ± 0.51
I will participate in such exercise program in the future	4.52 ± 0.51

Data presented in mean ± SD

#### Adoption 

A total of 100% adoption rate was achieved at the setting level. All seven senior activity centers with schedule availability that were approached agreed to participate in the study. However, the intervention was not conducted at two centers due to insufficient number of participants who met the study’s eligibility criteria.

#### Implementation 

At the provider level, among the total of 96 sessions conducted across four senior activity centers, two (2.1%) sessions had minor adaptations by the trainer. One session reduced the number of repetitions for one set of power component exercise and the other session prescribed different balance exercises of lower difficulty. At the participant level, the mean attendance rate attained was 87.5% among those who completed the program. Most (87%) participants achieved an attendance rate of at least 80%, and 17.4% (*n* = 4) of the participants completed all program sessions. On average, participants were found to complete 95.7% of the prescribed exercise program in the sessions they attended. Reasons for failing to complete the program include being late, feeling unwell, and leaving the session early. Seven (30.4%) participants achieved 100% participant fidelity rate.

## Discussion

The present study evaluated the effectiveness and implementation of a 12-week FPT program for pre-frail and frail older adults with low grip strength at their neighborhood senior activity centers. The FPT program significantly improved SPPB score of the participants. Evaluation of the intervention indicated good reach, effectiveness, adoption, and implementation outcomes. In support of our hypothesis, our study showed that an effective FPT program can be successfully implemented at local senior activity centers by local providers.

Performance of daily activities and life-threatening risks (e.g., falls) are more closely related to muscle power than strength in older adults (Cadore & Izquierdo, 2018). Structured muscle power training involving specialized equipment has been reported to be beneficial in improving physical function and muscle strength among frail individuals (Cadore et al., 2014; Zech et al., 2012). The results of the present study support such mode of exercise prescription and provided important evidence on translation by showing that a FPT program implemented in real-world settings can improve training specific functional performance in pre-frail/frail older adults. Our result strengthens available evidence that exercise interventions using simple equipment could be effective in improving physical performance or mitigating functional decline (Gill et al., 2002; Tan et al., 2018; Tarazona-Santabalbina et al., 2016; Westhoff et al., 2000). It is an important finding that such an exercise intervention can be implemented in local community settings as an effective option to complement what is currently available.

Significant interaction between group and time found for SPPB in the present study suggests that the intervention program was effective in improving physical function. Participants in the IG were found to exhibit small-moderate improvement in SPPB scores after the intervention. In contrast, SPPB scores of the CG did not change. The mean increase of 0.52 points in SPPB scores of IG met the recommended cut-off of 0.5 points in representing a small and clinically meaningful change in physical function among older adults (Perera et al., 2006). Examination of individual components revealed that the improvement in SPPB scores resulted specifically from improvement in repeated chair stand performance. This training specific improvement is not surprising. Previous studies reported that high velocity resistance training is effective in increasing repeated chair rise ability among older adults (Bean et al., 2009; Steib et al., 2010). In addition, the use of bodyweight as resistance in exercise programs to simulate daily living activities has been recommended to enhance functional capacity (Cadore et al., 2013). Hence, this interaction effect is likely attributed to the emphasis on high velocity muscle contraction and specificity of the program prescribed exercises to daily activities. Since repeated chair stand is a key feature of everyday activity, improvement in such function is likely to enhance quality of daily living.

While the program intervention improved frailty status and marginally improved TUG, the changes did not differ significantly from that in the CG. Given the small effect size, the lack of significance could be attributed to a low statistical power achieved. This was due to an unexpected and mandatory suspension of senior center activities and the study due to COVID-19, resulting in a smaller sample. Nevertheless, the results showed that the intervention program was at least as equally effective as the current exercise program offered at the senior activity centers. In contrast to functional performance, the FPT program did not elicit significant improvements in both HGS and KES among participants in the IG. This differs from previous report of increase in muscle strength among frail older adults with muscle power training (Cadore et al., 2014). The conflicting results may be attributed to the difference in intervention protocols between the studies. As the FPT program was designed to improve function performance, the exercise intensity may be inadequate to elicit significant strength gains. Similar results were reported in other studies of functional exercise interventions (Giné-Garriga et al., 2010; Manini et al., 2007).

Besides demonstrating effectiveness in physical outcomes, the results also showed that the FPT program is feasible in terms of reach, participation of frail older persons, participation, adoption, and implementation by local providers in actual community settings based on the RE-AIM framework (Glasgow et al., 1999). The intervention exhibited good reach with 98.4% of eligible individuals opting to join the study. Despite the drop-out rate of 14.8%, participants who completed the intervention showed good adherence with mean attendance rate of 87.5%. Although the post-intervention questionnaire was only administered to participants who completed the intervention, results indicated that these participants generally had positive experience in the community-delivered training program. In addition, the intervention showed good fidelity at both provider and participant level with minor but necessary deviation from the intended protocol. This showed that the exercise program was suitable for the targeted vulnerable population. Furthermore, the intervention had excellent adoption rate of 100% from the community senior activity centers approached. However, the findings also highlighted a potential challenge in which some centers have inadequate number of eligible participants to implement the program. Some spouses of participants wanted to enroll but did not meet the muscle weakness criteria. It is possible that these individuals could be involved either as volunteers or participants. Collectively, these outcomes could better inform relevant stakeholders in the delivery of such exercise programs in the community.

Senior activity centers receive government subsidy for their programs and operations and depending on their socio-economic status, members either pay a small fee or join for free. This study provides evidence that such a program to maintain function in frail participants warrants additional government funding. As centers do not have the appropriate manpower to conduct such a program, this service can be contracted to a local provider for cost sharing between centers. Due to COVID-19-related suspension of group activities at the senior centers, the provider has worked with some centers to offer FPT program via Facebook livestream (Gym challenge; https://empower.org.sg/onlinegymchallenge). A strategy to bridge the digital divide of some older adults has been to pair them up with others in their family or neighbors who can help with digital access and engagement with the live online training program. While not specifically measured in our study, the social engagement has been shown to be an important aspect of adherence to group training (Liu et al., 2019).

This study demonstrated that a community-delivered FPT program can be effective and feasible to reduce frailty and improve function in vulnerable older persons. However, it is important to acknowledge some limitations associated with the study. First, the study population consists of community-dwelling pre-frail and frail older adults and may not generalize to those of robust frailty status and frail older adults in the hospitals or nursing homes. In addition, readers should be cautious in generalizing the results given that the sample was largely women. However, this demographic profile is aligned with that of local senior community activity center membership of largely women (Thang, 2005; Wong et al., 2019). Second, due to the COVID-19 disruption of the study, we were unable to compare any post-program maintenance effects between the two groups. Given that physical function improvements induced by power training were reported to retain longer as compared to strength training (Zech et al., 2012), the longer term effects of the FPT program warrant further study.

## Conclusions

This study showed that community-delivered FPT program was a feasible intervention for frail older adults in their neighborhoods. Compared to normal physical exercise activities offered at local senior activity centers, the FPT intervention was associated with greater improvement in functional performance in pre-frail/frail participants. Positive implementation outcomes suggest that the program is promising for wider community translation.

## Supplementary Information

Below is the link to the electronic supplementary material.

Supplementary file1 (DOC 220 KB)
